# Recent survival trends in diffuse large B‐cell lymphoma––*Have we made any progress beyond rituximab?*

**DOI:** 10.1002/cam4.3237

**Published:** 2020-06-18

**Authors:** Narendranath Epperla, John L. Vaughn, Megan Othus, Abrahao Hallack, Luciano J. Costa

**Affiliations:** ^1^ Division of Hematology The Ohio State University Columbus OH USA; ^2^ Division of Hematology and Medical Oncology Weill Cornell Medicine New York NY USA; ^3^ Department of Biostatistics Fred Hutchinson Cancer Research Center Seattle WA USA; ^4^ Division of Hematology and Bone Marrow Transplantation Universidade Federal de Juiz de Fora BR USA; ^5^ Division of Hematology and Oncology Department of Medicine University of Alabama at Birmingham Birmingham AL USA

**Keywords:** diffuse large B‐cell lymphoma, incidence, population outcomes, relative survival rate, survival

## Abstract

**Background:**

Population‐based studies previously showed an improvement in overall survival (OS) for patients with diffuse large B‐cell lymphoma (DLBCL) who received chemoimmunotherapy with rituximab. However, there is limited data (especially at the population level) that show a similar trend in OS improvement, in the most recent time period. We hypothesized that survival for DLBCL patients diagnosed in the United States has continued to improve in recent years and intended to measure outcome improvements.

**Methods:**

Using the SEER‐18 registries, we compared the incidence and relative survival rates (RSRs) of DLBCL patients between 2002‐2007 and 2008‐2013 (availability of novel agents, broader use of autologous hematopoietic cell transplantation and improvement in supportive care). Multivariable Cox regression models were used to assess associations between the year of diagnosis and OS while controlling for age, gender, stage, and ethnicity.

**Results:**

There were a total of 53 439 patients with DLBCL who were diagnosed between 2002 and 2013. Of these, 25 810 were diagnosed during time period‐1 and 27 629 diagnosed during time period‐2. There was a slight decline in incidence of DLBCL (time period‐1 vs time period‐2), 7.75 (95% CI = 7.66‐7.84) vs 7.43 (95% CI = 7.34‐7.52) cases per 100 000 persons, respectively (*P* < .0001). Overall, there was a modest improvement in DLBCL RSRs, with 5‐year RSR improving from 61% (time period‐1) to 64% (time period‐2) and the improvement was noted across all subsets of patients. On multivariable analysis, patients diagnosed in time period‐2 had lower mortality relative to time period‐1 (HR = 0.87, 95% CI = 0.85‐0.89).

**Conclusions:**

Our study shows an improvement in the outcomes of DLBCL patients beyond the introduction of rituximab, although the magnitude of improvement is small. It will be interesting to see the impact of chimeric antigen receptor‐T cell therapy translating to population‐level survival in the next 5 years.

## Graphical abstract

There is a paucity of data at the population level on the overall survival trend in DLBCL patients in the contemporaneous time period. We show that although there has been a continuous improvement in the outcomes of DLBCL patients over the past decade, the magnitude of improvement is small, reflecting the modest progress made beyond rituximab in the treatment of DLBCL.

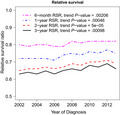

## 1 INTRODUCTION

Diffuse large B‐cell lymphoma (DLBCL) is the most common histologic subtype of non‐Hodgkin lymphoma (NHL) and accounts for approximately 25%‐30% of all lymphoid neoplasms in the developed world.^1^ Incidence increases with age (median age at presentation is 70 years) with a slight male preponderance.^1^

Population‐based studies previously showed an improvement in overall survival (OS) for patients with DLBCL who received chemoimmunotherapy with rituximab.^2^, ^3^ However, there is a paucity of data (especially at the population level) that shows a similar trend in OS improvement in the most recent time period. Although there were two recent studies updating population outcomes of DLBCL based on Surveillance, Epidemiology, and End Results (SEER) program they did not address this specific question. While one study^3^ reported the outcomes of DLBCL patients that precede the availability of novel agents; the other study^4^ was limited to the very elderly patients and is not reflective of the entire DLBCL patient population. We hypothesized that survival for patients diagnosed with DLBCL in the United States has continued to improve in recent years; and intended to measure outcome improvements and identify possible disparities.

## 2 MATERIALS AND METHODS

### 2.1 Patients and data source

We used the population‐based SEER‐18 registries to calculate the incidence, mortality, and relative survival rates (RSRs) of DLBCL in the United States for two consecutive time periods since the broad availability of rituximab in upfront DLBCL treatment. The two time periods were 2002‐2007 (time period‐1, early years after the adoption of rituximab) and 2008‐2013 (time period‐2: the introduction of novel agents, broader use of autologous hematopoietic cell transplantation [auto‐HCT] and improvement in supportive care). SEER collects cancer incidence (with a mandated case ascertainment of 98%), disease characteristics, treatment, mortality, and survival information from 18 geographic areas in the United States., representing 28% of the population. We identified DLBCL cases using the third edition of the International Classification of Disease for Oncology (ICD‐O‐3) histology codes 9679/3, 9680/3, 9684/3, 9688/3, and 9712/3 recorded between 2002 and 2013, who were older than 20 years of age at diagnosis, and in whom DLBCL was the first malignant neoplasm regardless of histologic subtype and survival follow‐up to the end of 2016. Primary central nervous system (CNS) lymphomas were excluded from the cohort by excluding ICD‐O‐3 topology codes C700‐701, C709‐729, and C751‐753. Cases diagnosed by autopsy or death certificate were excluded.

### 2.2 Data analysis and statistics

The SEER database contains information on age at diagnosis, year of diagnosis, sex, race/ethnicity, clinical stage based on the Ann Arbor system, vital status, and survival time. SEER collects limited information on the use of chemotherapy and radiation therapy, but not on HCT. For this analysis, age was categorized into two age groups of <65 years and 65 years and older and the stage was categorized into early (stage 1 and 2) and advanced (stage 3 and 4). We analyzed 3 main race/ethnicity subsets, non‐Hispanic whites (NHW), non‐Hispanic blacks (NHB), and Hispanics (of any race).

We calculated age‐adjusted incidence rates, age‐adjusted mortality rates, and RSRs with corresponding 95% confidence intervals using the rate function, incidence‐based mortality function, and survival function, respectively, in SEER*Stat 8.3.6. Age‐adjusted mortality rates were calculated using SEER cause‐specific death classification to capture deaths related to DLBCL. For this analysis, the cause of death is determined by cancer registries based on death certificates, and causes of death other than DLBCL are censored. We compared the annual percent change (APC) between the two time periods. APC was calculated by fitting a least squared regression line to the logarithm of the rates and calendar year was the single covariate. The coefficient from the two regression models was compared to evaluate if there was a difference in mortality rates between the two time periods. The definition of RSRs and the use of RSRs, as opposed to OS, have been explained in detail elsewhere.^5^, ^6^ Contrary to disease‐specific survival, RSRs are not influenced by the cause of death attribution. Standard statistical measures were used to compare continuous variables such as the Kruskal‐Wallis test for nonparametric data and Z‐test for the proportions. Multivariable Cox regression models were used to assess associations between the year of diagnosis and OS while controlling for age, gender, stage, and ethnicity. A two‐sided *P*‐value ≤ .05 was considered statistically significant. All analyses were conducted using IBM SPSS (version 22.0).

## 3 RESULTS

### 3.1 Patient characteristics

There were a total of 53 439 patients with DLBCL that were diagnosed between 2002 and 2013. Of these, 25 810 were diagnosed during time period‐1 and 27 629 diagnosed during time period‐2. The median age at diagnosis was 66 years in both the time periods. This is slightly lower than expected likely due to the exclusion of patients with prior malignant neoplasms. The median duration of follow‐up was 71 months (range, 0‐179 months) in time period‐1 and 44 months (range, 0‐107 months) in time period‐2. Patient characteristics of the study population stratified by time period‐1 and time period‐2 are outlined in Table 1.

**TABLE 1:** Baseline patient characteristics

Variable	Total (N = 53 439)	2002‐2007 (N = 25 810)	2008‐2013 (N = 27 629)
Age (in years)			
<65	25 470 (48%)	12 339 (48%)	13 131 (47%)
≥65	27 969 (52%)	13 471 (52%)	14 498 (53%)
Gender			
Male	28 975 (54%)	13 832 (54%)	15 143 (55%)
Female	24 464 (46%)	11 978 (46%)	12 486 (45%)
Race			
Non‐Hispanic Whites	37 589 (70%)	18 687 (72%)	18 902 (68%)
Non‐Hispanic Blacks	4116 (8%)	1994 (8%)	2122 (8%)
Hispanics	7039 (13%)	3075 (12%)	3964 (14%)
Others	4695 (9%)	2054 (8%)	2641 (10%)
Stage			
I‐II	23 978 (45%)	12 060 (47%)	11 918 (43%)
III‐IV	26 455 (49%)	12 205 (47%)	14 250 (52%)
Unavailable	3006 (6%)	1545 (6%)	1461 (5%)
Median duration of follow‐up of survivors in months, (range)	49 (0‐179)	71 (0‐179)	44 (0‐107)

### 3.2 Incidence

Diffuse large B‐cell lymphoma incidence (DLBCL cases per 100 000 persons) decreased from 7.75 (95% CI = 7.66‐7.84) in 2002‐2007 to 7.43 (95% CI = 7.34‐7.52) in 2008‐2013 (*P* < .0001). A decrease in the incidence rate was significant among both younger (<65 years, 4.14 to 3.92, *P* < .0001) and older patients (≥65 years, 24.53 to 23.73, *P* = .006), both males (9.18 to 8.88, *P* = .005) and females (6.54 to 6.2, *P* < .0001), NHW (7.98 to 7.61, *P* < .0001) and NHB (5.88 to 5.48, *P* = .02). Although there was a decrease in the incidence rate among Hispanics it was not significant (8.35 to 8.15, *P* = .35). While there was a significant decrease in the incidence rate among patients with early‐stage (3.61 to 3.20, *P* < .0001), there was an increase in the incidence among patients with advanced stage DLBCL (3.66 to 3.82, *P* = .0002) (see details in Table S1).

### 3.3 Mortality

Age‐adjusted mortality rates for DLBCL increased from 2002 to 2007 (APC = 10.6%), then decreased from 2008 to 2013 (APC = −0.66, *P* = .0193). Differences in mortality trends between 2002‐2007 and 2008‐2013 were statistically significant across all subsets of patients except those aged 20‐64 years and NHB patients (Table S2).

### 3.4 Survival

**FIGURE 1:**
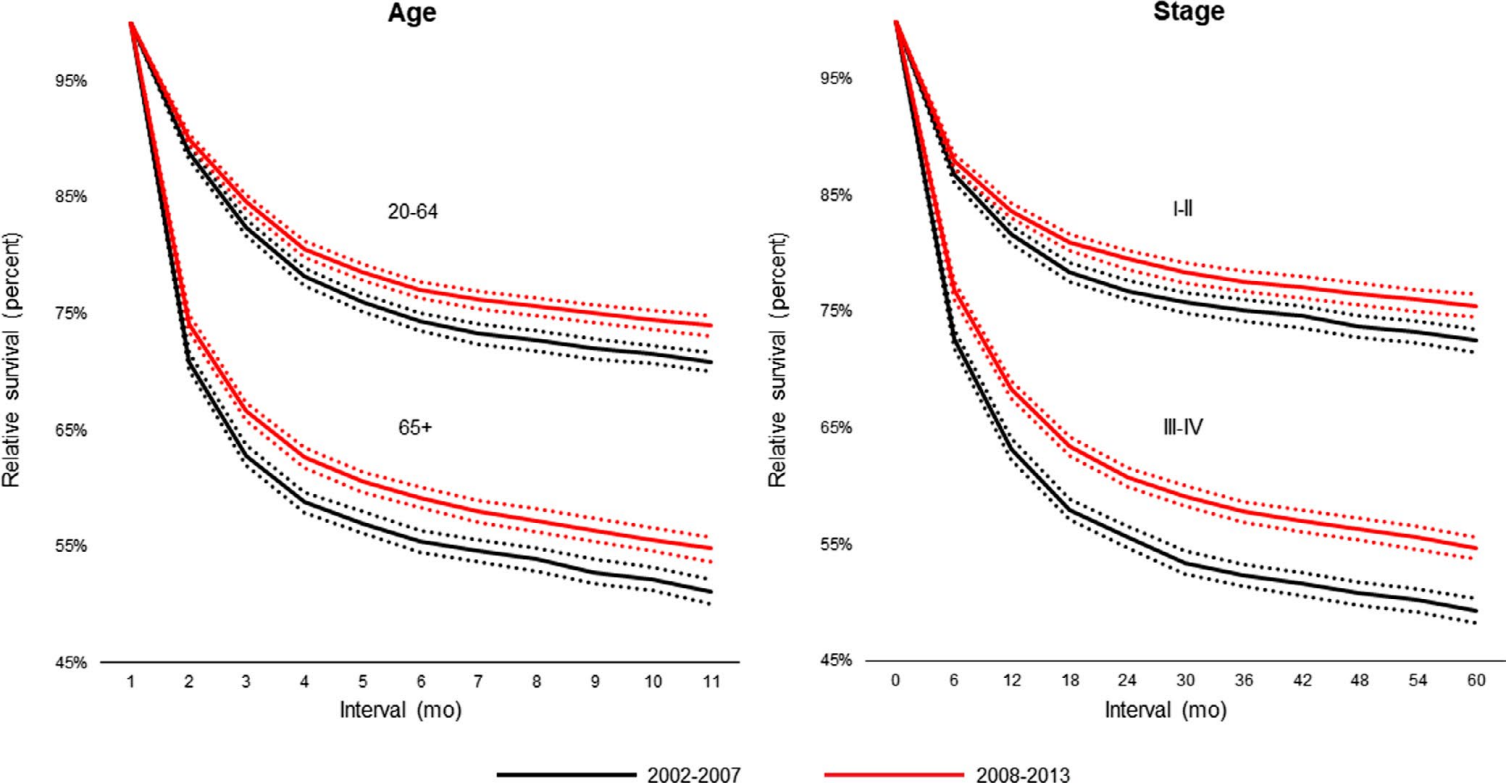
RSRs of DLBCL patients between 2002‐2007 and 2008‐2013 for age and stage

**FIGURE 2:**
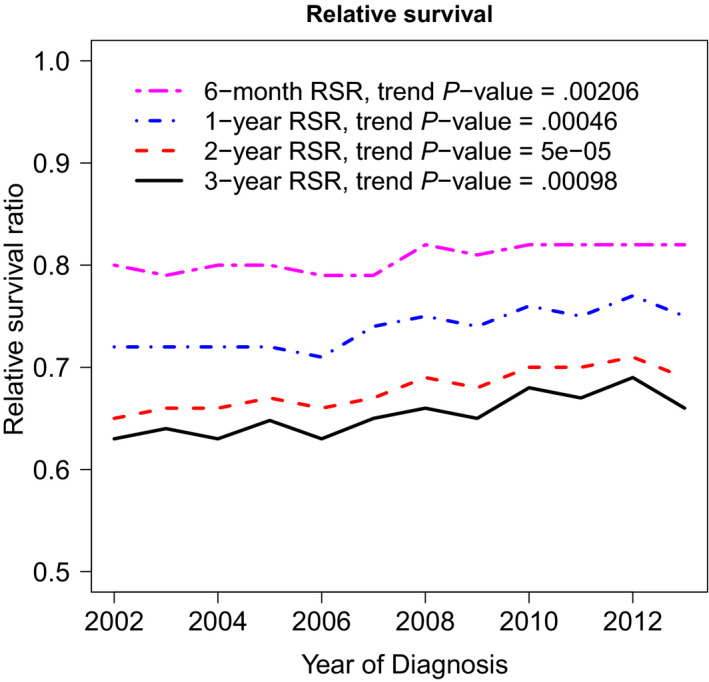
RSR trend between 2002 and 2012

Overall, there was a significant improvement in DLBCL RSRs, with 5‐year RSR (RSR‐5) improving from 61% to 64% between 2002‐2007 and 2008‐2013, respectively, *P* < .0001. Although improvement in RSR affected all subsets of patients (Table 2), the improvement was most evident in the first year after diagnosis and was more pronounced in patients with advanced stage (Figure 1). Figure 2 shows the temporal trend of RSRs over the study period (2002‐2013).

**TABLE 2:** RSR between two time periods, 2002‐2007 and 2008‐2013 by year

	RSR (95% CI)				
	RSR‐1	RSR‐2	RSR‐3	RSR‐4	RSR‐5
All patients					
2002‐2007	72.2% (71.7%‐72.8%)	66.2% (65.5%‐66.8%)	63.6% (63%‐64.3%)	62.1% (61.4%‐62.8%)	60.7% (60.1%‐61.4%)
2008‐2013	75.2% (74.7%‐75.7%)	69.2% (68.6%‐69.8%)	66.7% (66.1%‐67.3%)	65.4% (64.7%‐66%)	64% (63.3%‐64.7%)
*P*	<.0001	<.0001	<.0001	<.0001	<.0001
Age					
20‐64 y					
2002‐2007	82.4% (81.7%‐83.1%)	76% (75.2%‐76.7%)	73.3% (72.4%‐74.1%)	72% (71.1%‐72.8%)	70.9% (70%‐71.7%)
2008‐2013	84.6% (83.9%‐85.2%)	78.6% (77.9%‐79.3%)	76.2% (75.4%‐76.9%)	75% (74.2%‐75.8%)	74% (73.1%‐74.8%)
*P*	<.0001	<.0001	<.0001	<.0001	<.0001
≥65 y					
2002‐2007	62.8% (62%‐63.7%)	57% (56.1%‐58%)	54.6% (53.7%‐55.6%)	52.8% (51.8%‐53.9%)	51.1% (50.1%‐52.2%)
2008‐2013	66.6% (65.8%‐67.4%)	60.6% (59.7%‐61.4%)	58% (57.1%‐58.9%)	56.4% (55.4%‐57.4%)	54.8% (53.7%‐55.8%)
*P*	<.0001	<.0001	<.0001	<.0001	<.0001
Ethnicity					
NHW					
2002‐2007	72.9% (72.2%‐73.6%)	67.2% (66.5%‐67.9%)	64.8% (64%‐65.5%)	63.3% (62.5%‐64.1%)	61.9% (61%‐62.7%)
2008‐2013	75.7% (75.1%‐76.4%)	69.9% (69.2%‐70.6%)	67.5% (66.8%‐68.3%)	66.3% (65.5%‐67.1%)	65.1% (64.2%‐65.9%)
*P*	<.0001	<.0001	<.0001	<.0001	<.0001
NHB					
2002‐2007	67.5% (65.3%‐69.6%)	58.8% (56.5%‐61%)	56.5% (54.1%‐58.8%)	55% (52.6%‐57.3%)	54.2% (51.8%‐56.5%)
2008‐2013	72.6% (70.6%‐74.5%)	66.3% (64.2%‐68.4%)	63.5% (61.2%‐65.6%)	61.3% (59%‐63.6%)	59.8% (57.4%‐62.2%)
*P*	.001	<.0001	<.0001	<.0001	.0001
Hispanics					
2002‐2007	70.8% (69.1%‐72.4%)	64% (62.2%‐65.7%)	61.3% (59.4%‐63.1%)	59.5% (57.6%‐61.4%)	58.3% (56.4%‐60.2%)
2008‐2013	73.3% (71.8%‐74.7%)	67% (65.4%‐68.5%)	64.7% (63%‐66.2%)	63.1% (61.4%‐64.7%)	61.9% (60.2%‐63.6%)
*P*	.02	.008	.004	.003	.003
Stage					
I‐II					
2002‐2007	81.6% (80.8%‐82.3%)	76.8% (76%‐77.7%)	75.1% (74.2%‐76%)	73.7% (72.8%‐74.6%)	72.5% (71.5%‐73.5%)
2008‐2013	83.7% (83%‐84.4%)	79.5% (78.6%‐80.3%)	77.6% (76.7%‐78.5%)	76.5% (75.6%‐77.4%)	75.5% (74.5%‐76.5%)
*P*	.0001	<.0001	<.0001	<.0001	<.0001
III‐IV					
2002‐2007	63.2% (62.3%‐64.1%)	55.6% (54.7%‐56.5%)	52.3% (51.4%‐53.3%)	50.8% (49.8%‐51.8%)	49.3% (48.3%‐50.3%)
2008‐2013	68.3% (67.5%‐69.1%)	60.8% (59.9%‐61.6%)	57.8% (56.9%‐58.7%)	56.3% (55.4%‐57.2%)	54.7% (53.7%‐55.6%)
*P*	<.0001	<.0001	<.0001	<.0001	<.0001

On multivariable analysis (Table 3), patients diagnosed in time period‐2 had significantly lower mortality relative to time period‐1 (HR = 0.87, 95% CI = 0.85‐0.89). The risk of mortality significantly increased with age (HR, 1.53 per decade; 95% CI = 1.52‐1.54), male gender (relative to females, HR = 1.18, 95% CI = 1.15‐1.21), advanced stage (relative to early‐stage, HR = 1.63, 95% CI = 1.59‐1.67) and NHB and Hispanics (relative to NHW, HR = 1.26 [95% CI = 1.50‐1.64], and HR = 1.24 [95% CI = 1.12‐1.39], respectively).

**TABLE 3:** Multivariable analysis

Covariate	HR	95% CI	*P*‐value
Time period			
Time period‐1 (2002‐2007)	Referent		
Time period‐2 (2008‐2013)	0.87	0.85‐0.89	<.001
Age			
Age at diagnosis, decades	1.53	1.51‐1.54	<.001
Gender			
Female	Referent		
Male	1.18	1.15‐1.21	<.001
Stage			
I‐II	Referent		
III‐IV	1.63	1.59‐1.67	<.001
Stage missing	1.27	1.20‐1.33	<.001
Ethnicity			
Non‐Hispanic Whites	Referent		
Non‐Hispanic Blacks	1.26	1.50‐1.64	<.001
Hispanics	1.24	1.120‐1.39	<.001
Non‐Hispanic American Indian/Alaska Native	1.17	0.986‐1.39	.17
Non‐Hispanic Asian and Pacific Islander	1.08	1.03‐1.13	<.001

## 4 DISCUSSION

The advent of rituximab significantly improved outcomes in patients with DLBCL as demonstrated in clinical trials and population‐based studies.^2^, ^3^, ^7^, ^8^, ^9^, ^10^ However, it is unclear if there has been any improvement in outcomes beyond rituximab. Our study indicates that although OS for DLBCL continued to improve during the 15 years following the introduction of rituximab, this improvement has been modest, without any racial disparities as evidenced by the improvement of RSR‐5 across all ethnicities. Additionally, we noted that there was a significant decrease in the incidence of DLBCL over the last 6 years, a phenomenon previously described by others.^11^ Previously published population‐based survival of DLBCL patients reported outcomes for those diagnosed between January 1, 2000, and December 31, 2007.^3^ In our study, we have prolonged follow‐up (survival follow‐up to the end of 2016) and are able to examine the effect of more recent changes in DLBCL management in a population that is also more diverse.

In the past decade, the identification of high‐risk features (eg, c‐MYC gene rearrangement)^12^, ^13^, ^14^ has helped define aggressive subsets within DLBCL leading to more intensive treatment approaches for these patients. For the onset of the second time period, the year 2008 was chosen, as most of the data on novel agents for the treatment of *relapsed/refractory* DLBCL came after 2007.^15^, ^16^, ^17^, ^18^, ^19^, ^20^, ^21^, ^22^ In addition, broader use of HCT,^23^ improvement in supportive care, and identification of high‐risk subsets with the use of intensive chemotherapies became more prevalent during the most recent time period. Of note, the increase in the auto‐HCT seems to occur at a time when the incidence of DLBCL is decreasing, likely reflecting greater utilization of auto‐HCT at relapse. The finding of decreased incidence of DLBCL in the time period‐2 may be attributed to the decline in the incidence of HIV,^24^ HIV infections,^24^ and better control of HIV (related to the use of antiretroviral therapy)^25^ with few patients developing DLBCL.^26^, ^27^ The decrease in the incidence rate among patients with early‐stage, while an increase in the incidence rate among patients with advanced stage in the most recent time period may be reflective of *stage migration* due to the broader use of positron emission tomography (PET) scan at diagnosis.^28^

We found significant although *modest* gain in RSR‐5 for DLBCL patients in the time period‐2 with most of the improvement occurring in the first year after diagnosis (RSR‐1) and most pronounced in patients with advanced stage disease. To understand the possible factors contributing to this effect, we need to realize that despite the advent of rituximab, 40%‐50% of DLBCL patients fail to either achieve remission with rituximab‐based first‐line therapies or relapse after attaining a complete remission. Furthermore, only ~50% of the DLBCL patients who received second‐line salvage therapy demonstrate sufficient chemosensitivity to undergo auto‐HCT and ~50% of these patients who underwent auto‐HCT will subsequently relapse.^29^ We speculate that several factors could have contributed to the RSR‐5 improvement in the more recent time period such as the availability of novel agents,^15^, ^16^, ^17^, ^18^, ^19^, ^20^, ^21^, ^22^ broader use of auto‐HCT,^23^ improvements in supportive care including uniform growth factor support (reducing early infectious deaths), a lower proportion of HIV + patients, and improvements in outcomes of HIV + patients with DLBCL.^26^, ^27^ In addition, intensification of induction regimens for high‐risk subsets, the ability of older patients to receive targeted therapies, and greater use of CNS prophylaxis could have contributed to this improvement. Although the novel agents such as lenalidomide, bortezomib, ibrutinib, and antibody/drug conjugates have activity in *relapsed/ refractory* DLBCL, their contribution to the RSR gain is modest as they encompass only a small fraction of the entire DLBCL patient population. More importantly, the studies incorporating novel agents (lenalidomide/bortezomib/ibrutinib) in the frontline setting were all negative,^30^, ^31^, ^32^, ^33^ which could be one of the main reasons for the lack of a greater magnitude of survival improvement between the two time periods.

Our analysis is limited by the lack of granular data especially the specifics on treatment (chemotherapy and auto‐HCT). Also, SEER does not list LDH or ECOG performance status, thereby limiting the reporting of outcomes according to IPI strata. The stage‐specific assessment of survival over time periods may be subjected to the “Will Rogers” phenomenon^34^ and need to be interpreted with caution. Although we do not report the utilization rates of rituximab in time period‐1 vs time period‐2, a previously published study showed that most of the DLBCL patients (70%‐80%) received rituximab in the time period‐1 (2002‐2007).^3^ We acknowledge the lack of corroborative data on the utilization rates of novel agents in the time period‐2.

## 5 CONCLUSIONS

Our findings confirm a continuous improvement (over the past decade) in the outcomes of patients with DLBCL, however, they also highlight only a small magnitude of change at the population level. This reflects the little progress made beyond rituximab in the treatment of DLBCL, as evidenced by the negative randomized studies that utilized the novel agents in the frontline setting. It will be interesting to see the impact of chimeric antigen receptor‐T cell therapy translating to population‐level survival in the next 5 years.

## Conflict of Interest

The authors do not have any relevant conflict of interest pertaining to the manuscript.

## AUTHOR CONTRIBUTION

NE and LJC designed the study. NE, JLV, and LJC collected data. NE wrote the first version of the manuscript. NE, JLV, MO, and LJC analyzed the data. NE, JLV, MO, AH, and LJC edited the manuscript. All authors reviewed and approved the final manuscript.

## Supporting information

Table S1‐S2

## Data Availability

The data that support the findings of this study are available from the corresponding author upon reasonable request.
