# Supplementary material for: Recent survival trends in diffuse large B‐cell lymphoma––Have we made any progress beyond rituximab?
Source: Cancer Med. 2020 Jun 18;9(15):5519–25. doi: 10.1002/cam4.3237 (PMC7402846; doi:10.1002/cam4.3237)
Supplement: Supplementary file 1 — Table S1‐S2 [file CAM4-9-5519-s001.DOCX]

**SUPPLEMENTAL APPENDIX**

**Table of Contents 1**

**Incidence rate between time period-1 and time period-2:** Table S1 2

**Mortality trends between time period-1 and time period-2:** Table S2 3

|  | **Incidence rate*** | **SE** | **95% CI Lower** | **95% CI Upper** | **Incidence rate*** | **SE** | **95% CI Lower** | **95% CI Upper** | **P-value** |
| --- | --- | --- | --- | --- | --- | --- | --- | --- | --- |
| **All patients** | | | | | | | | | |
| Overall | 7.75 | 0.05 | 7.66 | 7.84 | 7.43 | 0.04 | 7.34 | 7.52 | <0.0001 |
| **Age** | | | | | | | | | |
| 20-64 | 4.14 | 0.04 | 4.06 | 4.21 | 3.92 | 0.03 | 3.85 | 3.99 | <0.0001 |
| 65+ | 24.53 | 0.21 | 24.12 | 24.94 | 23.73 | 0.2 | 23.34 | 24.11 | 0.006 |
| **Gender** | | | | | | | | | |
| Male | 9.18 | 0.08 | 9.02 | 9.33 | 8.88 | 0.07 | 8.74 | 9.03 | 0.005 |
| Female | 6.54 | 0.06 | 6.42 | 6.65 | 6.2 | 0.06 | 6.09 | 6.31 | <0.0001 |
| **Ethnicity** | | | | | | | | | |
| NHW | 7.98 | 0.06 | 7.86 | 8.09 | 7.61 | 0.06 | 7.5 | 7.72 | <0.0001 |
| NHB | 5.88 | 0.13 | 5.61 | 6.15 | 5.48 | 0.12 | 5.24 | 5.73 | 0.02 |
| Hispanics | 8.35 | 0.16 | 8.03 | 8.67 | 8.15 | 0.14 | 7.88 | 8.43 | 0.35 |
| **Stage** | | | | | | | | | |
| Stage 1-2 | 3.61 | 0.03 | 3.55 | 3.68 | 3.2 | 0.03 | 3.15 | 3.26 | <0.0001 |
| Stage 3-4 | 3.66 | 0.03 | 3.6 | 3.73 | 3.82 | 0.03 | 3.75 | 3.88 | 0.0002 |
| Unknown | 0.48 | 0.01 | 0.45 | 0.5 | 0.41 | 0.01 | 0.38 | 0.43 |  |

**Table S1: Incidence rate between time period-1 and time period-2**

Abbreviations: SE=standard error; CI=confidence interval; NHW= non-Hispanic whites; NHB= non-Hispanic blacks

*incidence rate is calculated based on DLBCL cases per 100,000 persons

**Table S2: Mortality trends* between time period-1 and time period-2**

|  | **2002-2007** | | | | **2008-2013** | | | |  |
| --- | --- | --- | --- | --- | --- | --- | --- | --- | --- |
|  | **APC*** | **SE** | **95% CI Lower** | **95% CI Upper** | **APC*** | **SE** | **95% CI Lower** | **95% CI Upper** | **P-value** |
| **All Patients** | | | | | | | | | |
| Overall | 10.64 | 0.054 | -0.30 | 22.79 | -0.66 | 0.56 | -3.49 | 2.26 | 0.02 |
| **Age** | | | | | | | | | |
| 20-64 | 11.42 | 0.14 | -5.62 | 31.54 | -0.84 | 0.55 | -4.31 | 2.76 | 0.08 |
| 65+ | 10.17 | 0.03 | 1.47 | 19.62 | -0.59 | 0.63 | -3.74 | 2.65 | 0.01 |
| **Gender** | | | | | | | | | |
| Male | 9.83 | 0.064 | -0.87 | 21.69 | 0.13 | 0.94 | -4.01 | 4.44 | 0.04 |
| Female | 11.26 | 0.047 | 0.20 | 23.54 | -1.23 | 0.23 | -3.59 | 1.19 | 0.01 |
| **Ethnicity** | | | | | | | | | |
| NHW | 10.36 | 0.05 | -0.30 | 22.15 | -0.40 | 0.76 | -3.74 | 3.05 | 0.02 |
| NHB | 11.34 | 0.09 | -2.64 | 27.32 | 0.28 | 0.90 | -5.34 | 6.23 | 0.08 |
| Hispanics | 11.74 | 0.01 | 3.80 | 20.29 | -1.93 | 0.56 | -10.02 | 6.89 | 0.02 |
| **Stage** | | | | | | | | | |
| I-II | 12.21 | 0.06 | -0.70 | 26.81 | -0.79 | 0.46 | -3.47 | 1.96 | 0.02 |
| III-IV | 10.35 | 0.05 | -0.23 | 22.05 | -0.16 | 0.92 | -4.29 | 4.15 | 0.03 |

Abbreviations: APC = annual percent change; SE=standard error; CI=confidence interval; NHW= non-Hispanic whites; NHB= non-Hispanic blacks

*Mortality trends are based on age-adjusted DLBCL-specific mortality rates

**Annual percent change was calculated by fitting a least squared regression line to the logarithm of the rates and calendar year was the single covariate. The coefficient from the two regression models was compared to evaluate if there was a difference in mortality rates between the two time periods.
